# Functional Characterization of MtrGSTF7, a Glutathione S-Transferase Essential for Anthocyanin Accumulation in *Medicago truncatula*

**DOI:** 10.3390/plants11101318

**Published:** 2022-05-16

**Authors:** Francesco Panara, Valentina Passeri, Loredana Lopez, Andrea Porceddu, Ornella Calderini, Francesco Paolocci

**Affiliations:** 1 Trisaia Research Center, Italian National Agency for New Technologies Energy and Sustainable Economic Development, (ENEA), 75026 Rotondella, MT, Italy; francesco.panara@enea.it (F.P.); loredana.lopez@enea.it (L.L.); 2 Institute of Biosciences and Bioresources, Consiglio Nazionale delle Ricerche, 06128 Perugia, PG, Italy; valentina.passeri@isafom.cnr.it (V.P.); francesco.paolocci@ibbr.cnr.it (F.P.); 3 Department of Agriculture, University of Sassari, Viale Italia, 39a, 07100 Sassari, SS, Italy; aporceddu@uniss.it

**Keywords:** *Medicago truncatula*, Tnt1 insertional mutants, flavonoids, gluthatione S-transferase, anthocyanin transport

## Abstract

Flavonoids are essential compounds widespread in plants and exert many functions such as defence, definition of organ colour and protection against stresses. In *Medicago truncatula*, flavonoid biosynthesis and accumulation is finely regulated in terms of tissue specificity and induction by external factors, such as cold and other stresses. Among flavonoids, anthocyanin precursors are synthesised in the cytoplasm, transported to the tonoplast, then imported into the vacuole for further modifications and storage. In the present work, we functionally characterised MtrGSTF7, a phi-class glutathione S-transferase involved in anthocyanin transport to the tonoplast. The *mtrgstf7* mutant completely lost the ability to accumulate anthocyanins in leaves both under control and anthocyanin inductive conditions. On the contrary, this mutant showed an increase in the levels of soluble proanthocyanidins (Pas) in their seeds with respect to the wild type. By complementation and expression data analysis, we showed that, differently from *A. thaliana* and similarly to *V. vinifera*, transport of anthocyanin and proanthocyanidins is likely carried out by different GSTs belonging to the phi-class. Such functional diversification likely results from the plant need to finely tune the accumulation of diverse classes of flavonoids according to the target organs and developmental stages.

## 1. Introduction

Flavonoids comprise a large group of secondary metabolites, including anthocyanins, proanthocyanidins (PAs), flavonols and isoflavones, which contribute in various ways to the growth and survival of plants [1,2,3]. Anthocyanidins are attractants for pollinators [4], protect plants against UV light damage and serve as defense molecules toward pathogens. Anthocyanins and simple monomeric and/or oligomeric PAs have been reported to protect against free radical injury and cardiovascular disease in humans [4]. PAs also act as antinutrients affecting both dietary protein and micronutrient availability in animals [5].

As other flavonoids, anthocyanin and the flavan 3-ols, catechins and epicatechins, the building blocks of PAs, are synthesised in the cytosol but accumulate within the vacuole, and therefore the efficiency of their transportation across the tonoplast affects their accumulation [6]. At least two different routes of flavonoid transport have been proposed: vesicle trafficking and transporter-mediated [7]. GSTs have been recognised as flavonoid transporters in a number of different taxa [6]. Moreover, they have been involved in the detoxification of a wide range of heterocyclic compounds (xenobiotics) by linking glutathione to the substrate giving rise to S-conjugates. To give an example, several classes of herbicides are glutathionated in the cytoplasm while the conjugate forms are accumulated within the vacuole [8,9,10].

GSTs form a large family of proteins, amounting to about 1% of total soluble leaf proteins [11]. Based on amino acid sequence similarity and exon-intron organization of genic models, plant GSTs have been divided in at least ten groups, including phi, tau, tetha, zeta, lambda, iota, dehydroascorbate reductase (DHAR), tetrachlorohydroquinone dehalogenase (TCHQD) and gamma subunit of the translation elongation factor 1B (EF1Bg) and hemerythrin [12,13]. Tau and phi GSTs are the most abundant in vascular plants [11]. The first demonstration that GSTs transport anthocyanins into vacuoles came from genetic analyses of maize *bz2*, a mutant line with bronze-coloured tissues and accumulating anthocyanins in the cytosol [14]. Since then, several other GSTs have been characterised as responsible for anthocyanin accumulation deficiency in plants. A seminal paper from Kitamura et al. [15] have demonstrated that *tt19*, a phi *GST* knockout, is impaired in both anthocyanin deposition in leaves and PA accumulation in seed testa. The double *tt19* phenotype has become an experimental model system to assay the activity of heterologous phi GSTs in either anthocyanin or PA transport [16,17,18]. For example, expression of MdGSTF6 in *tt19* background restored the accumulation of anthocyanin in leaves but not of PAs in seeds of *Malus domestica* [19]. Three phi GSTs have been functionally characterized in *Vitis vinifera* for their involvement in anthocyanins and PA accumulation [18]. Two of these, VviGST1 and VviGST3, were able to restore PA accumulation in seeds but not anthocyanin in leaves when constitutively expressed in *tt19* Arabidopsis plants. VviGST4, instead, restored both anthocyanin and PA accumulation in *tt19* background [18]. Bioinformatic analyses of protein ligand interactions suggested that these GSTs can bind glutathione, monomers of anthocyanins, PAs and flavonols. However, while the sites for GSH and flavonoids partially overlapped in the VviGST3 and VviGST1 proteins, they were well separated in VviGST4, similarly to the TT19 protein.

A modest amount of PAs in forage promotes an increased use of nitrogen derived from dietary protein and a reduction of pasture bloat [20,21,22]. When fed with forages rich in proteins, such as alfalfa or clover, plant species lacking PAs in their aerial portion, the pasture bloat phenomenon may occur in ruminants. To reduce bloating, surfactants able to break down foams or forages known to contain moderate levels of PAs can be added to the diet [21]. Both are costly options that determine a certain reluctance toward the use of alfalfa or clovers despite their excellent nutrient quality. *Medicago truncatula* has become the model forage species for genomic and functional studies and a large body of knowledge on flavonoid biosynthesis is now available on this species.

*M. truncatula* GST family includes up to 73 members which have been divided in eight classes with tau and phi being the most numerous ones [13]. Lineage specific expansion of gene family arose from large- or small-scale gene duplications followed by sub-functionalization. In this work, we analyze the function of *MtrGSTF7* a phi class GST. Although other *phi GSTs* are expressed in the same tissues of *MtrGSTF7*, the lack of *MtrGSTF7* transcript resulted in complete loss of anthocyanins in leaves, suggesting that this gene is essential for their accumulation.

## 2. Results

### 2.1. MtrGSTF7 Knockout Prevents Anthocyanin but Not Proanthocyanin Accumulation in M. truncatula Organs

**Figure 1:**
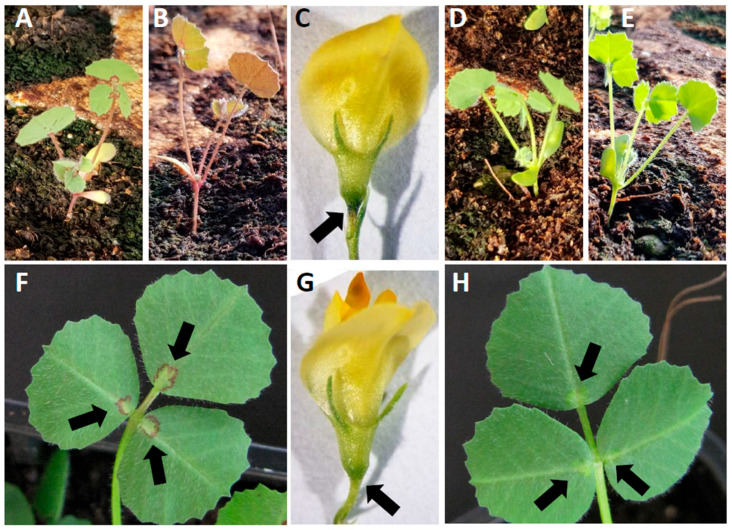
Pictures of wild -type plants (**A**–**C**,**F**) and R45 mutant line (**D**,**E**,**G**,**H**). Arrows in (**F**,**H**) point to the leaf blot (**F**) and to the corresponding position in R45 (**H**). Arrows in (**C**,**G**) point to the darkened region at the junction of pedicel and calyx (**C**) and to the corresponding position in R45 (**G**). Pictures (**A**,**B**,**D**) E were taken in a cold greenhouse where pigmentation in the aerial tissues was induced by low temperatures in the wild type (**A**,**B**), while no pigmentation was observable in R45 (**D**,**E**).

During a phenotypic screening of a *Tnt1* mutagenized collection [23], *M. truncatula* R108 lines R45 and R39 were noticed for the lack of leaf blots and red pigmentation on the aerial organs (Figure 1).

**Figure 2:**
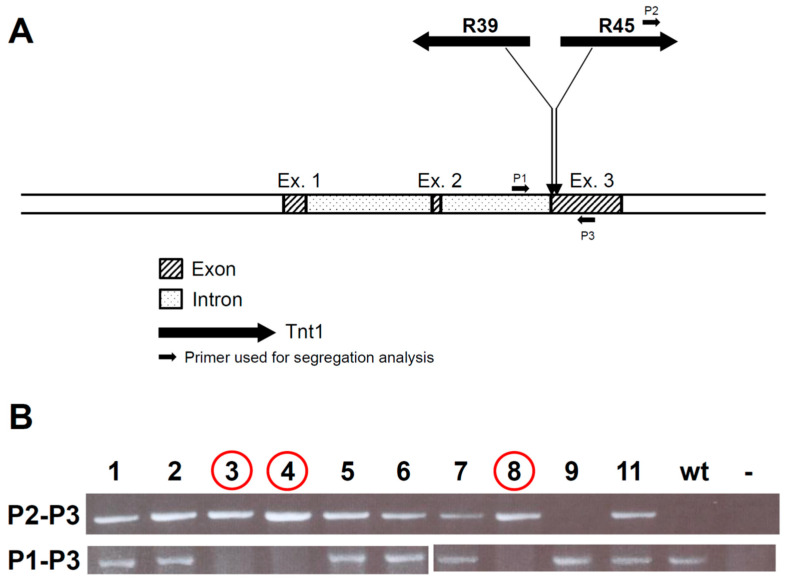
Intron-exon structure of *MtrunR108HiC_012760* corresponding to *MtrGSTF7* and position of *Tnt1* insertions in exon 3 in R39 and R45 mutant lines (**A**). Segregation analysis on 10 plants in the R45 T_1_ progeny. P1–P3 primed amplification fails in presence of the 5kbps *Tnt1* insertion. Plants 3, 4 and 8 (circled in red) showed the mutant phenotype (**B**).

*Tnt1* flanking regions from both lines were cloned by thermal asymmetric interlaced PCR (TAIL-PCR), and their genomic positions were identified within the R108 reference sequence [24]. It is noteworthy that one insertion from R45 and one from line R39 were mapped to close genomic positions, both within the *MtrunR108HiC_012760,* which encodes for the MtrGSTF7 phi class glutathione S-transferase protein [13]. Sequence tagged site (STS) markers were developed for each cloned insertion (see Figure 2A) and tested on sibs of the two mutant lines. Both STS markers tagging the *MtrGSTF7* insertions co-segregated with the phenotype according to mono-factorial recessive mutation models. These findings were also confirmed on progenies produced by selfed heterozygous sibs of R45 and R39 lines (Figure 2B).

**Figure 3:**
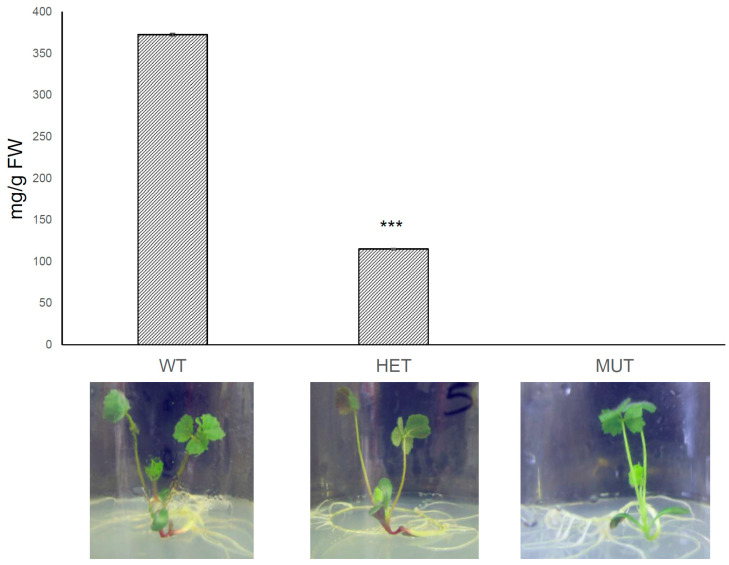
Anthocyanin content measured in leaves of plants cultivated in vitro on high sucrose (6%) containing medium. WT is wild-type R108. HET and MUT stands for heterozygous and homozygous referred to *Tnt1* insertion in *MtrGSTF7*. Anthocyanins could not be detected in MUT. Data indicate mean values ±SD of four replicates (*** *p* < 0.001).

Anthocyanins were undetectable by spectrophotometric analysis of leaves of both R45 and R39 homozygous individuals. A segregating progeny of an R45 heterozygous sib was cultivated in vitro on anthocyanin inducing medium (6% sucrose). Even under these conditions, we could not detect anthocyanins in leaves of R45 homozygous individuals, whereas individuals heterozygous for the insertions accumulated anthocyanins in leaves, though at levels significantly lower than wild-type sibs (Figure 3). R45 mutant sibs did not show qualitative differences for PAs when glandular trichomes and seed coats were stained with 4-(dimethylamino)-cinnamaldehyde (DMACA) (data not shown).

**Figure 4:**
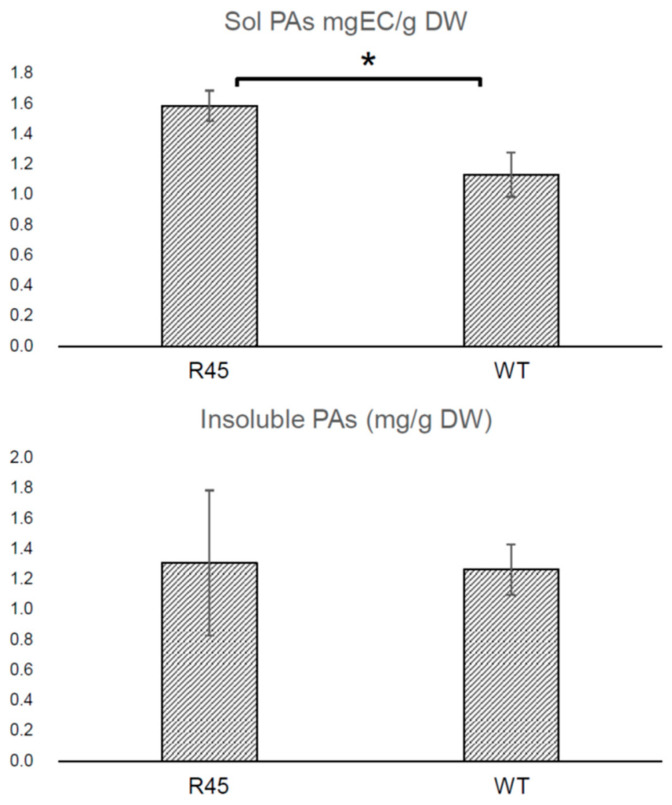
Contents of soluble and insoluble PAs measured in R45 and R108 (WT) seeds (* *p* < 0.05).

Notably, spectrophotometric analysis of extracts from seeds harvested from R45 homozygotes and heterozygotes showed a significant increase in soluble PAs compared to the wild-type seeds (*t*-test, *p* < 0.05), whereas the levels of insoluble PAs did not change (Figure 4).

**Figure 5:**
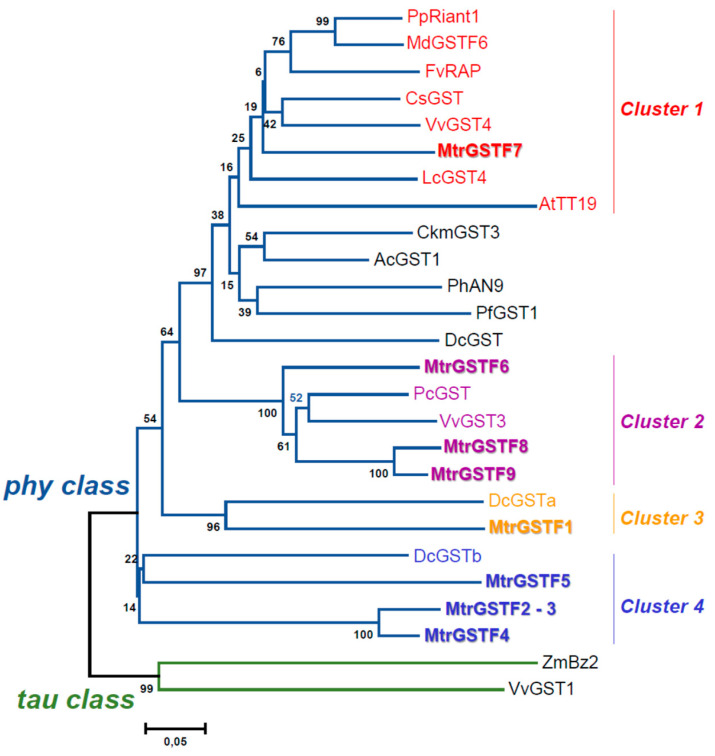
Phylogenetic tree with R108 MtrGSTFs and other GSTs involved in flavonoid transportation. The tree was constructed with the neighbor joining method (1000 replications of bootstrap test) and p-distance substitution model, pairwise deletion using the MEGA 6 program. PpRiant1 (*Prunus persica* ALE31199.1); MdGSTF6 (*Malus domestica* NP 001315851.1); FvRAP (*Fragaria vesca* XP 004288578.1); CsGST (*Citrus sinensis* ABA42223.1); VvGST4 (*Vitis vinifera* AAX81329.1); LcGST4 (*Litchi chinensis* ALY05893.1); AtTT19 (*Arabidopsis thaliana* OAO91277.1); CkmGST3 (*Cyclamen persicum* x *Cyclamen purpurascens* BAM14584.1); AcGST1 (*Actinidia chinensis* QCQ77644.1); PhAN9 (*Petunia x hybrida* CAA68993.1); PfGST1 (*Perilla frutescens* var. crispa BAG14300.1); DcGST (*Dianthus caryophyllus* BAM21533.1); PcGST (*Pyrus communis* ABI79308.1); VvGST3 (*Vitis vinifera* ABO64930.1); DcGSTa (*Dracaena cambodiana* ANH58194.1); DcGSTb (*Dracaena cambodiana*, ANH58192.1); ZmBz2 (*Zea mays* AAA50245.1); VvGST1 (*Vitis vinifera* AAN85826.1).

Previous studies have identified 9 phi GSTs in *M. truncatula* [13]. Based on sequence homology, we analysed the genetic distances among these proteins and phi GSTs characterised in other species (Figure 5).

*M. truncatula* phi GSTs were assigned to four clusters. MtrGSTF7 grouped in cluster I with GSTs essential for anthocyanin transport from the cytoplasm to the vacuole. The *Malus domestica* MdGST6 and the *V. vinifera* VVGST4 which complement both the anthocyanin and PA deposition in Arabidopsis *tt19* mutant belong to this cluster [19].

MtrGSTF6, MtrGSTF8 and MtrGSTF9 were in cluster 2 along with *V. vinifera* VvGST3, which complements PAs but not anthocyanin accumulation defect in *tt19* mutant [18]. The remaining phi GSTs from *M. truncatula* were positioned in two additional clusters (3 and 4), which showed the least distance from VvGST1 [18] and bz2 [14] tau GSTs.

### 2.2. Expression Analysis

Several studies have demonstrated that *phi GSTs* transcript levels are differentiated among organs and developmental stages. The gene expression atlas of *Medicago* organs [25] was interrogated for all available phi *GST*s probes. *MtrGSTF9* and *4* showed the highest levels of tissue specificity being readily detectable only in roots and almost not detectable in other organs. *MtrGSTF8* was expressed at high levels almost exclusively in seeds at the first developmental stages. *MtrGSTF7* and *MtrGSTF1* transcripts were readily detectable in aerial organs: *MtrGSTF7* was almost undetectable in seeds while a decreasing trend was observed for *MtrGSTF1*.

**Figure 6:**
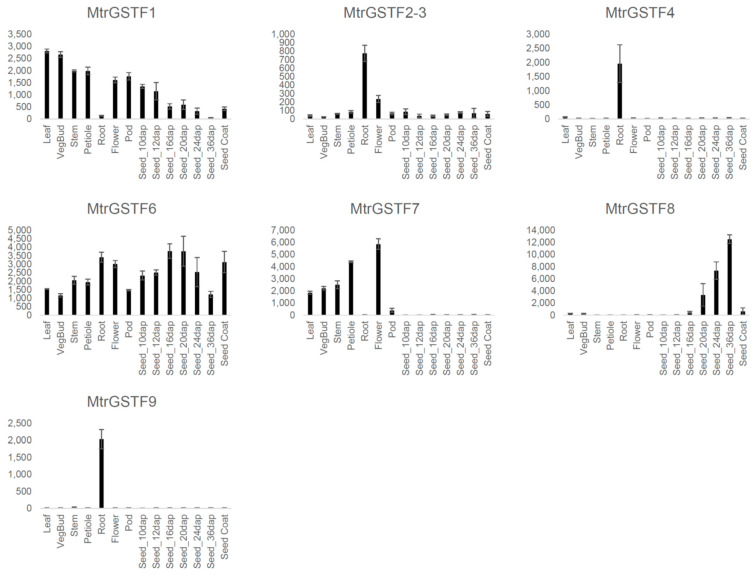
*MtrGSTFs*, expression in plant organs and in the seed coat.

In *M. truncatula*, high amounts of PAs accumulate in the seed coat. *Phi GST* expression data in seed coat indicate a high expression for *MtrGSTF6*, *MtrGSTF8* and *MtrGSTF1*, suggesting that these three *GST*s are possibly involved in PA accumulation in this tissue (Figure 6).

**Figure 7:**
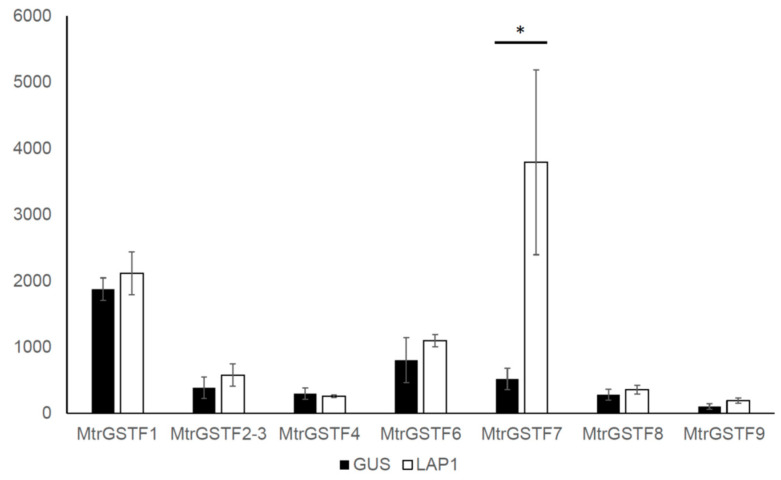
Expression of *MtrGSTFs* in leaves of 35S::LAP1 (LAP1), showing hyperaccumulation of anthocyanins, and 35S::GUS used as control (GUS). Asterisk indicates log_2_FC > 2.

Next, we analysed in silico the expression of *phi* GSTs in leaves of *M. truncatula* overexpressing the transcription factor LAP1 and showing hyperaccumulation of anthocyanins in leaves [26]. As shown in Figure 7, only *MtrGSTF7* was upregulated in these conditions, while the expression of other *GST*s was nearly unchanged.

**Figure 8:**
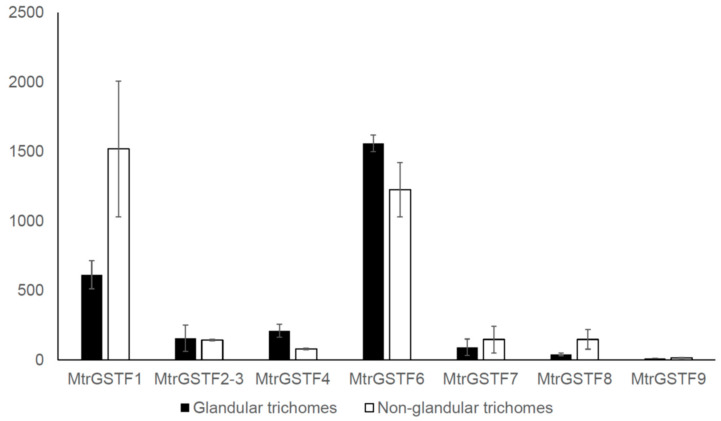
*MtrGSTFs* expression in glandular (black bars) and non-glandular trichomes (white bars).

Since glandular trichomes are known sites of PA accumulation, we interrogated the Trichome expression database [27] for *phi GST* accumulation. Figure 8 reports the expression levels of *M. truncatula phi GST*s in glandular and non-glandular trichomes. Higher expression was detected for *MtrGSTF1* and *MtrGSTF6* with log_2_FC value below 2 between the two types of trichomes. All other *MtrGSTFs* showed lower expression values. This was expected for *MtrGSTFs* with organ-specific expression in root and seeds. Interestingly, *MtrGSTF7*, which is highly expressed in leaves, showed low expression in trichomes.

**Figure 9:**
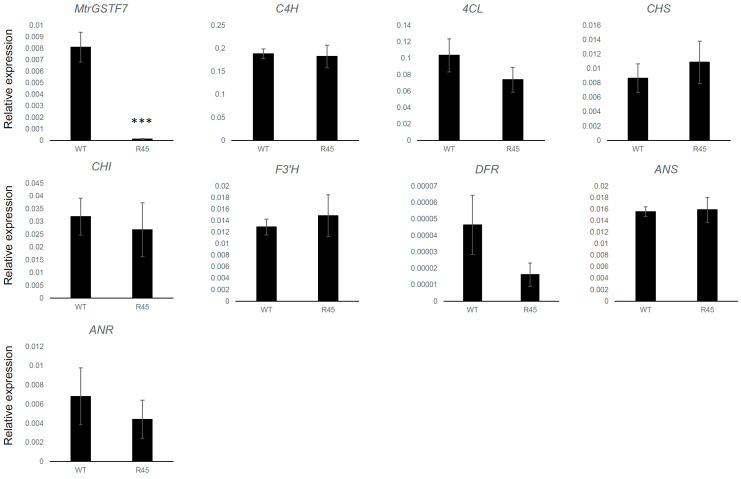
Expression levels of key genes of the phenylpropanoid pathway in *M. truncatula* leaves of wild-type R108 (WT) and mutant plants (R45) grown under controlled conditions. The relative expression of each gene is calculated using the [2^−(ΔCt)^] algorithm using actin as housekeeping gene. Significance of differences between means was determined by *t*-test (*** *p* < 0.001).

**Figure 10:**
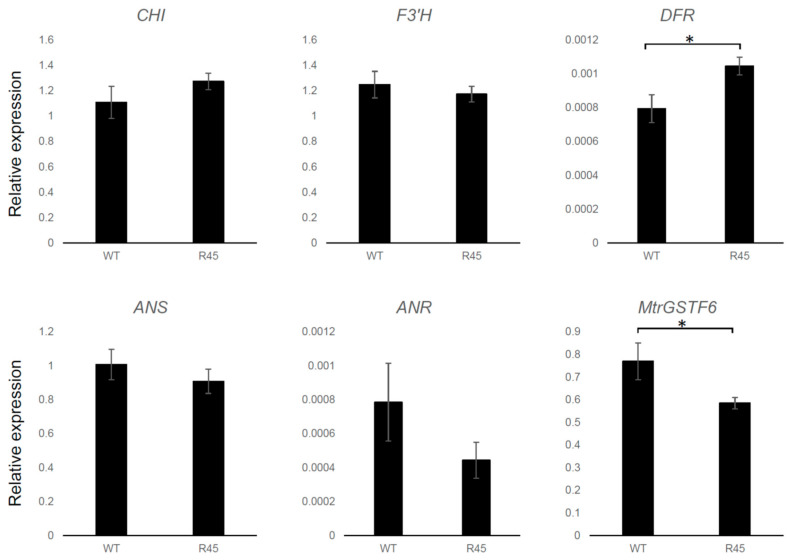
Gene expression in leaves of *M. truncatula* R108 (WT) vs. mutant plants (R45) grown in cold conditions. Expression levels are calculated as in legend of Figure 9 (* *p* < 0.05).

**Figure 11:**
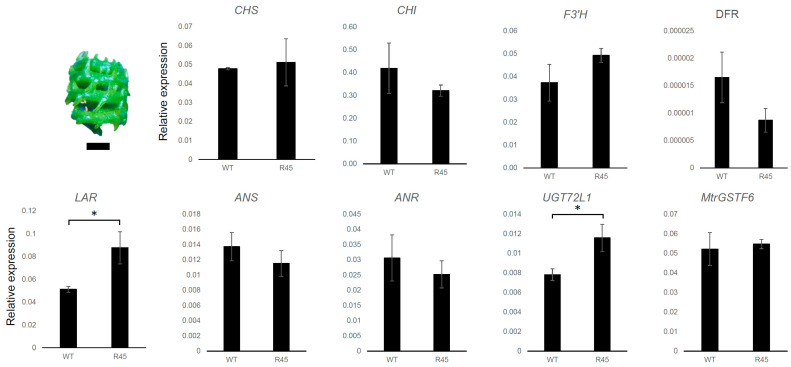
Gene expression in pods of *M. truncatula* R108 (WT) and mutant (R45). The stage of the sampled pods is shown (bar = 1 mm). Expression levels are calculated as in legend of Figure 9 (* *p* < 0.05).

A redirection of metabolic flux from anthocyanin to flavonol biosynthesis has been demonstrated in the Arabidopsis *tt19* mutant background [15]. It is noteworthy that the metabolic shift is not associated with a reduced expression of anthocyanin biosynthetic genes which are instead expressed at higher levels in *tt19* compared to wt plants. A similar picture was observed in *MtrGSTF7* mutant lines, in which the dramatic reduction in the *MtrGSTF7* transcripts levels was paralleled neither from that of early (*C4H*, *4CL*, *CHS*, *CHI* and *F3*′*H*) nor from that of late (*DFR*, *ANS* and *ANR*) key genes of the phenylpropanoid pathway when compared to wt plants grown under controlled conditions (Figure 9). However, when the same plants were grown under cold condition (cold greenhouse with average temperatures of 10 °C, see Figure 1A,B,D,E), an increase in the expression levels of *DFR* and a decrease in those of *MtrGSTF6* was detected in R45 leaves (Figure 10). Since R45 showed an increment in the levels of soluble PAs in the seeds, we decided to investigate the expression patterns of key genes as above along with that of *LAR* and *UGT72L1* in wt and R45 pods collected at an early stage (6 days after pollination) of their development when immature seeds therein stained positively with DMACA. As shown in Figure 11, the steady state mRNA levels were not statistically different for all the genes investigated except for *LAR* and *UGT72L1.* The former codes for the enzyme are not only responsible for the synthesis of catechin [28,29] but also for the control of the degree of PA polymerisation in that it promotes the accumulation of soluble rather than insoluble PAs [30]. The latter codes for an epicatechin-specific glucosyltransferase, which is expressed in the seed coat of *M. truncatula* [31].

### 2.3. Restoration of Anthocyanin Transport Deficiency of the Arabidopsis tt19 Mutant

**Figure 12:**
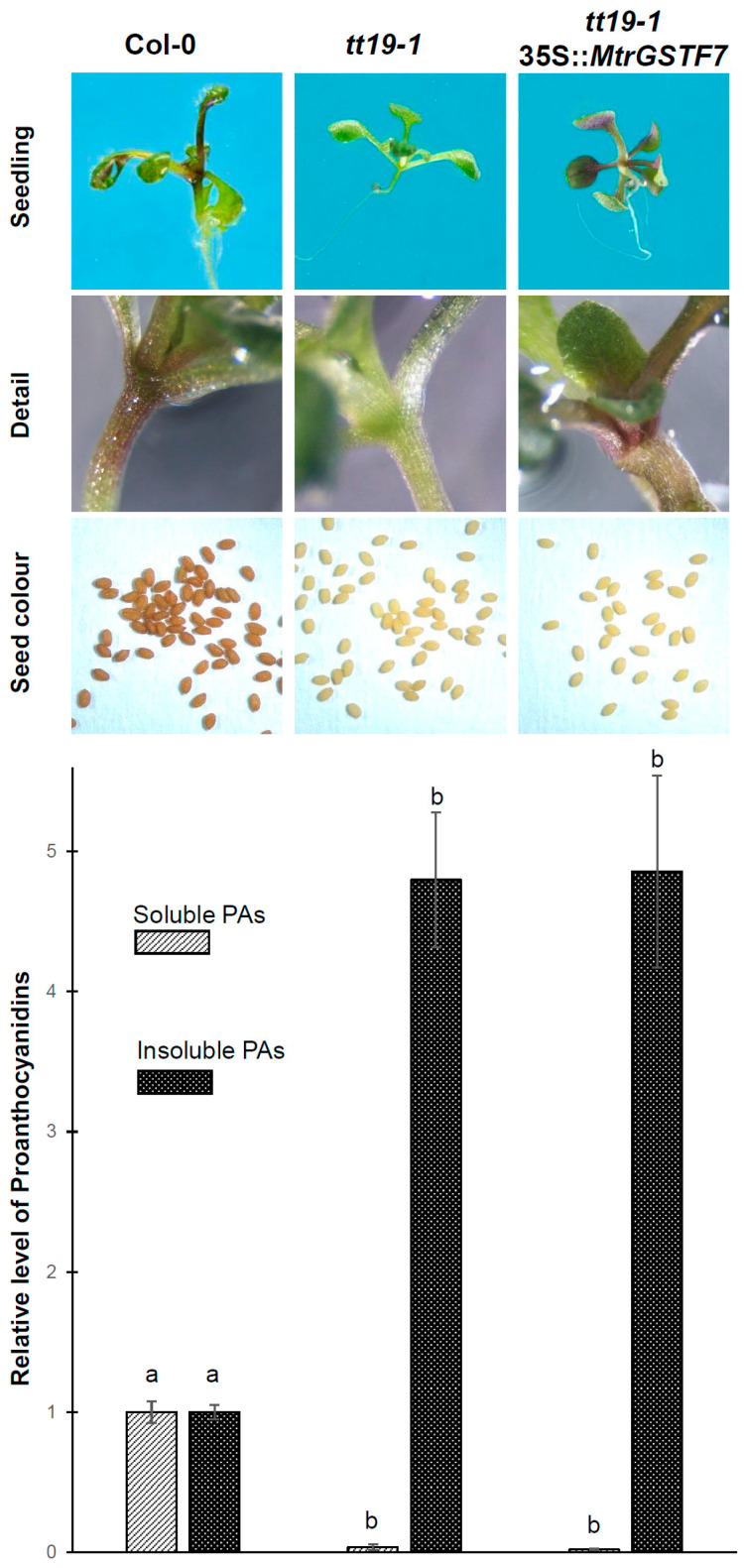
Functional complementation assay. From the top: seedlings; detail of anthocyanin colouration at the rosette base; seeds; relative content of soluble and insoluble PA in wt seeds vs. those from *tt19-1* mutant and *tt19-1* mutant transformed with the 35S::*MtrGSTF7* construct. Significant differences (*p* < 0.05) as determined by Tukey’s multiple comparisons test are indicated with different letters.

Arabidopsis *tt19* mutant is defective in anthocyanin accumulation in vegetative tissues and shows pale testa colour in the seeds [15]. To better elucidate MtrGSTF7 function in vivo, we adopted a functional complementation approach on *tt19-1* by generating transgenic *tt19-1* plants expressing *MtrGSTF7* under the constitutive promoter 35S from CaMV. Seedlings of transgenic and control plants were grown on high sucrose substrate to induce anthocyanin accumulation. As shown in Figure 12, the 35S::*MtrGSTF7* construct in *tt19* fully restored the anthocyanin accumulation in vegetative organs but had no effect on the levels of soluble PAs in seeds (Figure 12).

## 3. Discussion

Anthocyanins are part of the flavonoid family, and they are the final products of a specific branch leading to the formation of flavonols, phlobaphenes and proanthocyanidins as well [32]. This class of metabolites confers resistance to several stresses in plant species via ROS signalling [33]. The biosynthesis of anthocyanins is extensively characterised, and both biosynthetic and regulatory genes have been identified in several plant species [34] showing a high degree of conservation. Concerning the transport of anthocyanins to the vacuole, two main mechanisms have been proposed, one using membrane transporters, and the second one involving the formation of vesicles which will merge with the tonoplast. In both models, the transport is assisted by GST. In particular, GST of the phi group are those involved in anthocyanin transport, with GSTs also having a plethora of different roles [35]. Biochemical assay has proved the binding of GST to anthocyanin in a few species, including Arabidopsis tt19; the conservation of the anthocyanin binding function was proved for GST from several other species by complementation analysis of the *Arabidopsis tt19* mutant. Among these are FL3 from carnation [36], PfGST1 from *Perilla frutescens* [37], VvGST1 and VvGST4 from grapevine [18,38], CkmGST3 from Cyclamen [39], CMGSTF12 from *Camelina sativa* [40], LcGST4 from *Litchi chinensis* [16], PpRiant1 from peach [41], FvRAP from strawberry [42], IbGSTF4 from sweet potato [43], MdGSTF6 from apple [19] and AcGST1 from kiwifruit [44].

Here, we report on the cloning and functional characterization of MtrGSTF7. *M. truncatula* functional genomics via mutant analysis has proved very powerful in understanding several aspects of plant biology of legumes, with particular focus on nodulation [45] and biosynthesis of secondary metabolites [46]. In this context, the presence of a leaf red blot is a useful marker to detect mutations in the anthocyanin pathway. In our work, the visual screening of a *Tnt1* mutagenized *M. truncatula* collection [23] led to the original identification of two mutants lacking the anthocyanin pigmentation of the leaf typical of the R108 genotype. The anthocyanin content remained negligible even after sucrose induction in in vitro grown plants of the mutant for MtrGSTF7 compared to the wild-type R108. The cloning of the gene responsible for the mutation undertaken by TAIL-PCR analysis of the transposon flanking regions led to the identification of MtrGSTF7. The two independent knock-out mutants, though lacking the ability to accumulate anthocyanins, are able to store PAs in both seed coat and glandular trichomes. Expression analysis by qRT-PCR of key biosynthetic genes of the phenylpropanoid pathway in leaves of plants grown under controlled conditions, while confirming a dramatic decrease in the levels of *MtrGSTF7* in the mutant line, did not show any significant difference between this and wt for all the other genes considered. Conversely, under cold conditions, differences emerged between R45 and wt plants, namely the upregulation of *DFR* in R45 and that of *MtrGSTF6* in wt leaves. Although both these results call for an in depth characterisation of both transcriptional profile and function of these genes, they let us argue that the block of the anthocyanin pathway due to the knocking out of *MtrGSTF7* causes a reprogramming of the metabolic pathway in leaves only under environmental conditions that promote flavonoid biosynthesis. Accurate targeted metabolomic analyses by HPLC-MS would help us to address this issue.

A thorough *M. truncatula* GST gene family characterisation has revealed the presence of nine phi GSTs with partially overlapping expression patterns. It is noteworthy that while the expression of *MtrGSTF2-3* is mainly root-specific and that of *MtrGSTF8* is concentrated in the last stages of seed development, the other *M. truncatula GSTF*s show overlapping patterns of expression in vegetative tissues. However, the observation that *MtrGSTF7* knockout totally abolishes the anthocyanin accumulation in vegetative tissues demonstrates that other *M. truncatula* phi GSTs, although being co-expressed with *MtrGSTF7*, are unable to functionally complement it. Several hypotheses could explain such a conclusion. First, other phi GSTs have a non-redundant function, for example they are not able to bind anthocyanin. Second, other GSTs are engaged in anthocyanin transport downstream of MtrGSTF7. One possibility would be that MtrGSTF7 could transform anthocyanin to a form recognisable by other GSTs. Enzymatic studies in *Petunia* and *Arabidopsis* have excluded that enzymatic modification of anthocyanin mediated by GSTs is needed for their binding and transport. Other studies have demonstrated that anthocyanin can also be transported by other transporters, such as MATE2 [47], and that, differently from MtrGSTF7, their knockout does not impair completely anthocyanin accumulation. These data lend support to the hypothesis of anthocyanin transport as a sequence of events, with MtrGSTF7 working at the early stages of this sequence. At the same time, MtrGSTF7 mutant seeds do not show loss in PA content, highlighting the specific involvement of MtrGSTF7 in the transport of one subclass of flavonoid, the anthocyanin. Interestingly, only *MtrGSTF7* expression notably increases upon overexpression of the MYB transcription factor LAP1, with LAP1 being an inducer of anthocyanin synthesis and accumulation in *M. truncatula* and *M. sativa* [26]. The other *phi GSTs* expressed in leaves do not show significant expression changes in *LAP1* overexpressing leaves. At the same time, the expression of *MtrGSTF7* is low in glandular trichomes, which accumulate PAs compared to whole leaf. Other *GST*s, such as *MtrGSTF1* and *MtrGSTF6,* showed higher expression in trichomes. These GSTs are also expressed in other sites of preferential PA accumulation (e.g., seeds), in which *MtrGSTF7* expression is barely detectable. All these data suggest that *MtrGSTF7* expression is associated with anthocyanin but not with PA accumulation. Better still, the increase in the levels of soluble PAs in the seeds of R45, coupled to the increase in the steady state levels of *LAR* and *UGT72L1* genes in the pods at early stage of R45 with respect to those from wt, are all evidence suggesting that knocking out of *MtrGSTF7* causes a flux diversion from anthocyanin towards PAs in *M. truncatula* reproductive organs. In turn, these results pave the way to future time course analyses to compare the metabolic and molecular profiles between R45 and wt developing seeds.

The complementation experiment of *Arabidopsis tt19* mutant showed that MtrGSTF7 is able to rescue the accumulation of anthocyanins in plant tissues but does not restore the presence of soluble PAs in the seeds. Thus, the transport mechanism in which MtrGSTF7 is involved is similar to what happens in Arabidopsis but limited to the anthocyanins. The *tt19* complementation assay is able to discriminate between GST transporters of anthocyanins and GST transporters of both anthocyanins and PAs. For instance, AN9, PpGST, LcGST4, Bract1, RAP, ScGST3, MdGSTF6, GhGSTF12, LhGST and PpGST1 only complement anthocyanin accumulation in vegetative tissues [16,19,25,41,42,48,49,50,51,52]. Differently, heterologous expression of *Camelina sativa* CMGSTF12, grape VviGST4, radish RsGST1, tea CsGSTa, CsGSTb and CsGSTc in Arabidopsis *tt19* mutant not only complements the anthocyanin accumulation in vegetative tissues but also complements the brown pigmentation in the seed coat [17,18,40,44]. Therefore, in *M. truncatula,* either a fine subfunctionalisation between GSTs involved in the transport of terminal branches of the flavonoid pathway occurs, or the GST responsible for the transport of both compounds has not been identified yet. Embracing the latter hypothesis, we note that the *M. truncatula* genome harbours several *phi* type GSTs, some of which, namely *MtrGSTF1*, *6* and *8*, have high expression in seeds. Further analysis, based on mutant characterisation, needs to be undertaken to unravel the function and organ specificity of the above mentioned *M. truncatula* genes in flavonoid transport under different environmental conditions. Finally, we note that while the writing of the current manuscript was underway, the paper by Wang et al. [17] concerning the functional characterisation of MtrGSTF7 as an anthocyanins specific transporter has been published. The present study not only reinforces but also provides additional evidence with respect to the above-mentioned study about the functional role of MtrGSTF7.

## 4. Materials and Methods

### 4.1. Plant Material

*M. truncatula* plants, both wild-type and *Tnt-1* mutants, were grown in three different conditions: (a) a cold containment greenhouse in the autumn-spring period, (b) under controlled conditions in a growth chamber at 23 °C under a 16/8 h (light/dark) photoperiod, in 2.5-L pots with commercial loam mixed 10% with expanded clay provided with irrigation and (c) in vitro at 23 °C under a 16/8 h (light/dark) photoperiod on SHM2/2 medium [53] with and without 50 g/L of additional sucrose (final concentrations 1% and 6% respectively). *A. thaliana* plants, Columbia and *tt19-1* mutant [15], were grown in a growth chamber under the same conditions used for *M. truncatula*. Samples were collected from plants and immediately frozen in liquid nitrogen. Frozen samples were stored at −80 °C until analysis.

### 4.2. Quantification of Anthocyanins and PAs

DMACA staining was used for qualitative estimation of PA accumulation plant organs as well as the analyses of soluble and insoluble PAs were performed according to [54]. Anthocyanin extraction and quantification were carried out as reported in [55].

### 4.3. Identification of the Mutated Gene

Tnt1 insertions in R45 and R39 mutants were recovered using a TAIL-PCR protocol as described in the *M. truncatula* handbook [56]. TAIL-PCR products were inserted in pGEM-T easy vector and cloned in *E. coli* XL1-blue cells. Transformed colonies were analyzed by PCR and sequenced by Sanger method with T7/Sp6 primer. Insertion sites sequences were analysed by Blast on the *M. truncatula* genome, and their position on available genome assembly was assigned. DNA from *M. truncatula* plants was extracted with GenElute Plant Genomic DNA Miniprep Kit (Sigma Aldrich, St. Louis, MO, USA) according to the manufacturer’s protocol. Segregation of Tnt-1 insertions was analyzed by PCR using primers spanning 100–200 bp in 5′ and in 3′ of the insertion site used in combination with the primer Tnt2rev (5′-GCA CAT GCC TAA TAC TTC TTC-3′) on Tnt-1 LTR.

### 4.4. RNA Isolation and cDNA Synthesis

RNA was extracted by Spectrum Plant Total RNA kit (Sigma–Aldrich, St Louis, MO, USA). After isolation, RNA was treated with DNase (Sigma–Aldrich) according to the supplier’s instructions, and the absence of any DNA contamination verified by the null PCR amplification of the RNA preps in the presence of the ITS1 (5′-TCC GTA GGT GAA CCT GCG G-3′) ITS4 (5′-TCC TCC GCT TAT TGA TAT GC-3′) primer pair as reported in [57] Three micrograms of RNA were reverse transcribed in the presence of Maxima H Minus Reverse Transcriptase (Thermo Scientific, Milan, Italy) and 100 pmol of random hexamers (Euroclone, Milan, Italy) according to the supplier’s instructions.

### 4.5. Gene Expression Analysis

#### 4.5.1. Real-Time PCR

A primer pair specific to each gene tested was designed with the help of OligoExpress Software (Applied Biosystems, Waltham, MA, USA). The primers are given in Table 1.

An aliquot of 2 µL of 1:10 diluted cDNA was used in the PCR reaction, which was performed using the BlasTaq 2X qPCR Mater Mix (ABM, Richmond, Canada) and carried out in an ABI PRISM 7300 SDS apparatus (Applied Biosystems) according to the following cycling parameters: an initial step at 95 °C for 3 min, followed by 40 cycles each, including a step at 95 °C, 15 s and a step at 60 °C, 1 min. A melting curve was added after each run. For each gene, four technical replicates were amplified. The PCR efficiency for each primer pair was tested as reported in [58]. Gene expression quantification was calculated using the (2^−ΔCt^) method as reported in [59] with *actin* as a reference gene. Three biological replicates were analysed, and the significance of the differences between mean expression values was analysed by *t*-test.

#### 4.5.2. In Silico Expression Analysis

*MtrGSTF*s expression was investigated by analyzing data available for two microarray experiments on plant tissues and seed coat [25,31]. *M. truncatula* gene expression data was downloaded from the *Medicago* Gene Expression Atlas web server [60] currently available at https://medicago.toulouse.inrae.fr/GEA, accessed on 25 February 2022 [61]. Expression data in glandular and non-glandular trichomes was downloaded from the TrichOME database currently available at https://trichome.zhaolab.org/trichomedb/, accessed on 10 December 2021 [27].

### 4.6. Phylogenetic Analysis

Sequences of GST proteins related to flavonoid transport were retrieved from the GenBank database. Accession numbers are shown in Figure 5 caption. *M. truncatula* GSTs of the phi class in the R108 genotype were retrieved based on previous genome wide analysis [13] by Blastp of A17 (*M. truncatula* v4.01 genome) proteins on R108 v1.0 genome. Proteins were aligned using ClustalW and a phylogenetic tree was constructed with the neighbor joining method (1000 replications of bootstrap test) and p-distance substitution model, pairwise deletion using the MEGA 6 program.

### 4.7. Arabidopsis tt19 Complementation

The ORF of MtrGSTF7 was PCR amplified from leaves cDNA of *M. truncatula* cv. R108 obtained by standard protocols. Primer GST_ATG_FW (5′-ATG GTG GTG AAA GTT TAT GGT TCA-3′) and GST_TAA_REV (5′-TTA CTT AGC CAA TTC CTT CAA C-3′) were used. The PCR product was cloned into RBCTA cloning vector (RBC Biosciences, Taiwan) according to the manufacturer’s instructions. Plasmids were isolated from positive colonies with an Innuprep plasmid mini kit (Analytik Jena, Germany) and sequenced by Sanger methods with an ABI3130 sequencing machine. Sequences were verified by Blast analysis with the available genomic resources of *M. truncatula*; the correct clone was used for sub-cloning into the plant binary vector pBI121 by substituting the GUS gene (XmaI-SacI fragment) with the GST gene obtaining the plasmid pBI121-GST7, which was verified by sequencing with primers 35sFW (5’-CTA TCC TTC GCA AGA CCC TTC-3’) and M13(-21) forward. The pBI121-GST7 plasmid was transferred into *Agrobacterium tumefaciens* GV3101 cells by electroporation. Colonies were verified by PCR, and the correct colony was stored with glycerol at −80°C and used for plant transformation via the floral dip method [62]. The Arabidopsis mutant *tt19-1* [15] was used for transformation. Transgenic plants were selected on MS plates added with kanamycin 50 mg/L, two independent homozygous lines for a single T-DNA insertion were obtained in T3 generation and seeds were used for further phenotypic analysis.

## Figures and Tables

**Table 1:** List of primers employed in qRT-PCR assays.

Gene	Forward Primer (5′-3′)	Reverse Primer (5′-3′)	N° Accession
*Actin*	TCAATGTGCCTGCCATGTATGT	ACTCACACCGTCACCAGAATCC	JQ028731.1
*4CL*	AGGAGGCAACCAGATCGACCAT	CAAACGATCGACGACAAATAAGAATCC	XM_013610757.3
*C4H*	CGATATCCCAGCCGAGAGCAA	AAGAACCGTTCGGGCCTGAAT	HM627322.1
*CHS*	GGTTCAGACCCATTACCACAAGTTGA	TGTAAGCCCGACTTCACGAAGGT	XM_003601599.4
*CHI*	GTAGCCATTTGGAAGTCTCTTGGGA	ATAGAGGAGCCTGGTGGAAATGTTT	XM_003592720.4
*F3’H*	GTCTTGGTCTTAGGATGGTTCAACTTCTTA	CTCTGTAAGGTGAGCCCATATGCTTCA	XM_013602525.3
*DFR1*	GGCTATGTGTCTTTATGTGTGTTTCTGTG	AGTTTGGGCTTCAACAGGATTGC	AY389346.1
*ANS*	AAGAAGCTGGTGGAATGGAAGAG	GGTTGAGGGCAAATTGGGTAG	EF544389.1
*ANR*	GTCGGGCTCATATTTTTGTGGC	GAAACTTTGCAAGCTCGGGAACACTG	AY184243.1
*UGT72L1*	CACTGGCTTGGAGCTTCTATTTG	CAGCCCGGTACTTTGATAGGC	EU434684.1
*GSTF7*	CAGTTGTAGAGGATGGTGATTTCAGA	ACCACGGTCTGCATACTTTGTT	MtrunR108HiC_012760
*LAR*	GAGGTTTTTGCCTTCAGAATTTGG	GGGTGGAGGAAGCTGTGATGG	BN000703.1

## Data Availability

All references to publicly archived datasets were reported in the Section 4 of the manuscript.
